# Bioinformatic and Machine Learning Applications in Melanoma Risk Assessment and Prognosis: A Literature Review

**DOI:** 10.3390/genes12111751

**Published:** 2021-10-30

**Authors:** Emily Z. Ma, Karl M. Hoegler, Albert E. Zhou

**Affiliations:** Department of Dermatology, University of Maryland School of Medicine, Baltimore, MD 21230, USA; ezma2005@hotmail.com (E.Z.M.); hoegler.karl@gmail.com (K.M.H.)

**Keywords:** melanoma, melanoma genomics, bioinformatics, machine learning, deep learning

## Abstract

Over 100,000 people are diagnosed with cutaneous melanoma each year in the United States. Despite recent advancements in metastatic melanoma treatment, such as immunotherapy, there are still over 7000 melanoma-related deaths each year. Melanoma is a highly heterogenous disease, and many underlying genetic drivers have been identified since the introduction of next-generation sequencing. Despite clinical staging guidelines, the prognosis of metastatic melanoma is variable and difficult to predict. Bioinformatic and machine learning analyses relying on genetic, clinical, and histopathologic inputs have been increasingly used to risk stratify melanoma patients with high accuracy. This literature review summarizes the key genetic drivers of melanoma and recent applications of bioinformatic and machine learning models in the risk stratification of melanoma patients. A robustly validated risk stratification tool can potentially guide the physician management of melanoma patients and ultimately improve patient outcomes.

## 1. Introduction

Cutaneous melanoma is the most aggressive form of skin cancer and the fifth most common cancer in the United States [1]. The incidence of cutaneous melanoma has been rising in the past few decades, with over 100,000 new cases diagnosed in the United States each year [1]. Despite recent advancements in advanced melanoma therapy, including targeted therapy (e.g., BRAF/MEK inhibitors) and immunotherapy (e.g., PD-1 inhibitors), there are over 7000 melanoma-related deaths each year in the United States, as the most advanced stage melanoma patients have recurrence after initial therapy [1,2,3].

The major risk factors for cutaneous melanoma formation are ultraviolet (UV) exposure and genetic susceptibility. UV-induced DNA damage and oxidative stress can cause the malignant transformation of melanocytes [4]. A family history of melanoma is a strong risk factor for the disease, which has led to the significant growth of melanoma genomics research in the past two decades [5].

The bioinformatic analysis of genomic data has been widely used to identify potential genetics and signaling pathways associated with melanoma pathogenesis and metastasis. More recently, bioinformatic analyses, including machine learning, are increasingly utilized to predict prognosis, risk stratify, and ultimately inform personalized treatment in cutaneous melanoma.

We conducted a literature review within PubMed and Google Scholar to provide an overview of bioinformatic and machine learning applications in melanoma prognostics and risk stratification. Given the massive catalog of bioinformatics and machine learning studies in the field of melanoma genomics and risk stratification, we attempt to summarize the currently established key drivers of melanoma that have utilized bioinformatics in its discovery. We also provide an overview of key findings, algorithms, and the predictive accuracy of recent studies applying bioinformatic and machine learning algorithms to melanoma risk stratification.

## 2. Bioinformatics in Melanoma Genomics

A melanoma is a heterogenous disease with numerous genetic determinants. Bioinformatic tools have been widely used to help understand the genetic drivers of melanoma and identify patient subgroups by specific genetic mutations to inform the management and development of therapies.

**Figure 1:**
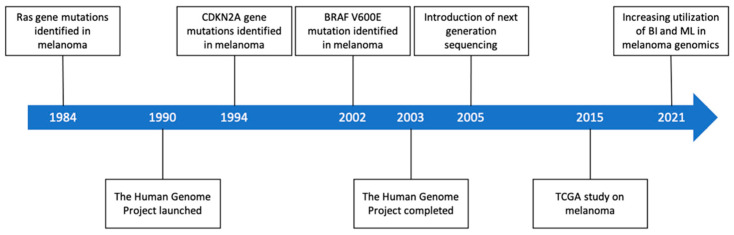
Key advances in melanoma genomic research. BI: bioinformatics, ML: machine learning.

Ras genes and CDKN2A were the earliest gene mutations identified in melanoma in the 1980s and 1990s (Figure 1) [6,7]. Ras genes are proto-oncogenes that are frequently mutated in cancers which encode a family of small G proteins, while CDKN2A encodes tumor suppressor proteins [8].

In 2002, one of the first genomic studies identified mutations in BRAF, a regulator of cell survival, in 65% of malignant melanomas [9], which led to the development of BRAF inhibitors for BRAF mutant metastatic melanoma [10,11].

The arrival of next generation sequencing (NGS) in the early 2000s precipitated the profiling of the full melanoma genome [12]. Since then, whole-exome sequencing (WES) has characterized mutations in NF1, ARID2, PPP6C, rAC1, SNX31, TACC1, and STK19 related to melanoma development [13,14]. In 2015, the Cancer Genome Atlas Skin Cutaneous Melanoma (TCGA) used WES to confirm previously identified melanoma mutations in BRAF, NRAS, CDKN2A, TP53, and PTEN [15]. TCGA also identified MAP2K1, IDH1, RB1, and DDX3X mutations in melanoma [15]. Figure 1 summarizes the key milestones in melanoma genomic research.

Recent whole-genome analyses of melanoma has also identified different mutated genes in cutaneous, acral, and mucosal melanoma, and highlighted mutations in the TERT promoter [16]. The TERT gene encodes the catalytic subunit of telomerase, an enzyme complex that regulates telomere length [16]. Additional genomic changes observed include changes in c-KIT, c-MET, and EGF receptors, and in MAPK and PI3K signaling pathways, which are important pathways for cell proliferation and survival [8].

The introduction of the high throughput analysis of biological information, particularly next-generation sequencing, has led to the rapid growth of genomic data [17]. As new genomic databases grow, additional genetic regulators of melanoma formation and progression are expected to be characterized in the future and potentially inform melanoma management.

## 3. Bioinformatics and Machine Learning in Melanoma Risk Assessment

Despite clinical staging guidelines, predicting the prognosis of melanoma is challenging due to its heterogenous nature. Bioinformatic tools have been widely used to analyze NGS data and help identify potential mutations associated with melanoma pathogenesis [18]. More recently, there have been increasing applications of bioinformatic analysis in melanoma risk stratification and the prediction of prognosis to inform treatment. Since the approval of systemic adjuvant therapies for stage III and stage IV melanoma, these therapies are now widely used following the resection of advanced melanoma. However, these systemic therapies are associated with frequent grade 3 or 4 adverse events, and are costly [19,20,21,22,23]. 2021 National Comprehensive Cancer Network (NCCN) guidelines currently do not recommend adjuvant therapy in stage I and II patients [24]. Patients with stage II melanoma have a 12% to 25% 10-year melanoma-specific mortality rate, and some stage II patients have worse survival than stage III patients [25,26]. As such, accurate prognostic tools to predict the probability of recurrence and survival are needed to risk stratify to better identify appropriate candidates for adjuvant treatment and level of surveillance.

### 3.1. Gene-Expression Profiling

The gene expression profiling of stage IV melanomas identified molecular subtypes with unique gene signatures that were correlated with different clinical outcomes [27]. This finding led to the development of a proprietary 31-gene expression profile (GEP) assay (Castle Biosciences) used to categorize the high- versus low-risk of metastases within five years of melanoma diagnosis [28,29]. One of the goals of 31-GEP testing was to determine the intensity of treatment and follow-up for melanoma patients.

The clinical utility and performance of 31-GEP has varied, and needs to be further validated in prospective studies [30]. Zager et al. analyzed 523 primary melanoma tumors using 31-GEP and reported that 31-GEP identified 70% of stage I and II patients who ultimately developed distant metastasis [31]. Similarly, Gastman et al. found that 31-GEP accurately identified high-risk patients who are likely to recur or die of melanoma in low-risk subgroups (e.g., sentinel lymph node-negative disease, stage I and IIA) [32]. A meta-analysis reported that 31-GEP performance varied, and was a better predictor of recurrence in stage II disease than in stage I [33]. However, a separate study suggested that there is limited cost-benefit of 31-GEP utilization in stage IIIA melanoma due to the limited survival benefit of this tool for this patient subgroup [34]. Given the lack of clear evidence that 31-GEP improves outcomes in melanoma, an established prognostic tool is still needed to accurately identify high-risk patients.

### 3.2. Current Bioinformatics in Melanoma Risk Assessement

A bioinformatic analysis of genes and biomarkers has not only been used to help identify genes associated with melanoma survival and mortality, but also to predict melanoma metastasis and prognosis (summarized in Table 1).

Several recent studies constructed protein-protein interaction (PPI) networks to identify hub genes in melanoma. Sheng et al. constructed a PPI network to analyze differentially expressed genes (DEGs) from the Gene Expression Omnibus (GEO) database [35]. The study identified DGS3, DSC3, PKP1, EVPL, IVL, FLG, SPRR1A, and SPRR1B as potential biomarkers that predict the metastases of cutaneous melanoma [35]. Another study constructed a PPI network from melanoma gene expression data from UCSC Xena and GEO and found FOXM1, EXO1, KIF20A, TPX2, and CDC20 as genes associated with reduced overall survival [36]. Results from Wang et al. indicated that high CD38 expression could be a diagnostic marker for melanoma, and found that higher CD38 expression levels resulted in improved survival probabilities compared to lower expression levels [37].

An analysis of miRNA expression from 59 melanoma metastases identified 18 miRNA signatures that were overexpressed and correlated with longer post-recurrence survival [38]. Furthermore, the study identified six miRNA signatures that were predictors of survival of stage III patients independent of American Joint Committee on Cancer (AJCC) staging [38].

Sentinel lymph nodes (SLNs) regulate anti-tumor immune responses, so Farrow et al. hypothesized that SLN gene expression could predict a recurrence risk in melanoma [43]. Immune-related genes from SLN biopsies were used to create a multivariate regression model to predict recurrence-free survival [39]. Twelve genes, including immune checkpoint TIGIT, accurately predicted RFS, and therefore could potentially inform patient selection for adjuvant therapy [39]. Several other prognostic biomarkers were identified with Cox regression analyses, including pre-operative circulating tumor DNA that have the potential to further enrich the stage IIIA population for high-risk adjuvant therapy candidates [42,47].

A logistic regression analysis was used to create a nomogram that predicted the probability of a positive SLN in melanoma based on tumor characteristics, such as tumor thickness, Clark level, ulceration, site, and patient sex and age [51]. The nomogram predicted the presence of SLN metastasis more accurately than the AJCC staging system and has been externally validated by three separate institutions [54,55,56].

### 3.3. Machine Learning in Melanoma Risk Asessement

Machine learning is the application of computer algorithms with the aim to optimize the predictive accuracy of the algorithm [57,58]. Machine learning algorithms are based on pattern recognition and are designed improve its behavior based on data or experience, without additional human intervention. These algorithms can be powerful tools to assist humans in the analysis of large, heterogenous data sets, such as genomic data sets.

Machine learning research in dermatology has been primarily focused in developing image recognition tools for the binary classification of malignant melanoma [59]. Recently, there are a growing number of machine learning studies that aim to risk stratify and predict prognosis in melanoma, with several models outperforming the current risk classification tools available (summarized in Table 1). Various machine learning algorithms were employed in the studies we reviewed, with neural networks, a support vector machine, and random forest classifier models as the more commonly utilized algorithms. Several studies were able to achieve an AUROC over 0.8, or accuracy greater than 80%, though there were no clear associations between the machine learning algorithm used and accuracy achieved. We do not compare the predictive abilities of these studies, as the models aimed to predict different outcomes.

Gene expression datasets from GEO and TCGA were used to construct a PPI network that identified 798 genes associated with melanoma metastasis [50]. These genes were used as variables in a support vector machine (SVM) classifier that had a metastasis prediction accuracy ranging from 96% to 100% [50]. A separate study used gene expression data from 754 thin- and intermediate-thickness primary cutaneous melanomas to train logistic regression models to predict the presence of SLN metastases from molecular, clinical, and histologic variables. The study found that models using clinicopathologic or gene expression variables were outperformed by a model that included molecular variables along with clinicopathologic predictions (i.e., Breslow thickness and patient age) [40]. Arora et al. also incorporated clinicopathologic variables in their machine learning models and found that models using clinicopathological features (e.g., Breslow thickness, N staging, M staging, ulceration status) outperformed GEP-based profiles and AJCC staging in predicting melanoma prognostics [39].

Several studies have utilized machine learning to analyze large RNA datasets and identify correlations with melanoma prognosis with high degrees of accuracy. Yang et al. used multiple machine learning algorithms to analyze melanoma samples from TCGA. The study hypothesized that six long non-coding RNA (lncRNA) signatures may regulate the MAPK, immune and inflammation-related pathways, the neurotrophin signaling pathway, and focal adhesion pathways [52]. The six lncRNA signatures were identified and used in a machine learning classifier that risk-stratified melanoma patients with 85% accuracy [52]. A separate study of transcriptomic data from 478 primary and metastatic melanoma, nevi, and normal skin samples identified six novel associations between the activation of metabolic molecular signaling pathways and the progression of melanoma [49]. A differential expression analysis of primary tumors from 205 RNA-sequenced melanomas revealed 121 metastasis-associated gene signatures which were then used to train machine learning classification models. The machine learning models better predicted the likelihood of metastases than models trained with clinical covariates or published prognostic signatures [53]. The analysis of RNA transcriptome data from cutaneous melanoma from Huang et al. found 16 m5C-related proteins that (e.g., USUN6, NSUN6) were also predictors of melanoma prognosis [45].

Mancuso et al. analyzed levels of selected cytokines with machine learning to classify stage I and II melanoma patients with a high and low risk of metastasis. The study found that cytokines IL-4, GM-CSF, and CDC with the Breslow thickness best predicted melanoma metastasis [48].

Johannet et al. used deep learning on histology specimens with clinicodemographic variables to predict low versus high risk of progression after immunotherapy in advanced melanoma [46]. A separate computation pathology-based cell classification algorithm demonstrated that a high ratio of lymphocytes to all lymphocytes within the stromal compartment and a high ratio of stromal cells to all cells correlated with a poor survival in melanoma [53]. Histology slides from primary melanoma tumors with known SLN metastasis were used to train a machine learning model to predict SLN status, though the model achieved 61% accuracy and was not clinically relevant [41].

## 4. Conclusions

Cutaneous melanoma is a genetically heterogenous disease with many patient subgroups associated with different outcomes. There are currently no melanoma risk stratification tools that have been well validated and widely used. Bioinformatic analyses, particularly machine learning, have been internally validated to accurately risk stratify melanoma patients. However, bioinformatic tools will need to be externally validated to have clinical utility. Bioinformatic and machine learning analyses are growing rapidly in the field of melanoma, and we anticipate that continued research in melanoma risk stratification tools can potentially change future patient management and outcomes.

## Figures and Tables

**Table 1:** Summary of major studies in bioinformatic and machine learning risk stratification of melanoma.

Publication	Methods	Key Finding(s)	Performance	Data
Arora et al. 2020 [39]	Multiple machine learning algorithms (e.g., SVM ^1^, decision tree, random forest)	Machine learning model based on clinicopathologic variables outperformed model based on GEP profiles or AJCC ^1^ staging in predicting OS ^1^		RNA expression data of cutaneous melanomas (CMs) (*n* = 458) from TCGA ^1^
Bellomo et al. 2020 [40]	Machine learning logistic regression model	Epithelial-to-mesenchymal transition and melanosome function genes were associated with SLN ^1^ metastasis; model combining clinicopathologic and gene expression variables better predicted SLN metastases than model with clinicopathologic or gene expression variables	AUROC ^1^: 0.82 (clinicopathologic and gene expression model)	Gene expression data of primary CMs (*n* = 754)
Brinker et al. 2021 [41]	Artificial neural network (ANN)	ANNs trained with H&E images not matched to SLN status had AUROC of 62% and may not be clinically relevant to predict SLN status	AUROC: 61.8% (matched), 55.0% (unmatched)	Primary melanoma with positive SLN H&E slides (*n* = 291)
Cheng et al. 2015 [42]	Multi-variate Cox regression analysis	BRAF and MMP2 were prognostic biomarkers for stage I/II, while p27 is a biomarker for stage III/IV		Primary (*n* = 148) and metastatic (*n* = 106) CMs
Farrow et al. 2021 [43]	Multi-variate Cox regression analysis	12 genes predicted RFS ^1^; increased *TIGIT* expression and decreased *CXCL16* correlated with improved RFS		RNA samples (*n* = 62) from SLN biopsies
Garg et al. 2021 [44]	Random forest classifier	Machine learning models trained with 121 metastasis associated genes performed better in predicting regional lymph node metastasis than models trained with clinical trained with clinical covariates or published prognostic signatures	P_AUROC_: 7.03 × 10^−4^ (combined model)	RNA data of primary CMs (*n* = 204)
Huang et al. 2021 [45]	Decision-tree algorithm (XGBoost)	5-methylcytosine (m5c) signatures were used to predict CM prognosis; NSUN6 may be a marker for CM progression		Transcriptomic data of CMs (*n* = 4761) from TCGA
Jiang et al. 2021 [36]	GO ^1^ and KEGG ^1^ enrichment analysis, PPI network analysis	Identified 435 DEGs ^1^; *FOXM1*, *EXO1*, *KIF20A*, *TPX2*, and *CDC20* were associated with reduced OS		Gene expression data of CMs from UCSC Xena (*n* = 322) and GEO (*n* = 45)
Johannet et al. 2021 [46]	Deep convolutional neural network (DCNN)	Machine learning algorithm trained with histology and clinicodemographic variables predicted immunotherapy response (PFS ^1^) in advanced melanoma patients with AUC ^1^ of 0.800	AUC: 0.800	Advanced melanoma patients (*n* = 121)
Jönsson et al. 2010 [27]	Unsupervised hierarchical clustering, two-group significance of microarray analysis (SAM), support tree analysis	Four distinct subtypes with unique gene signatures are associated with different prognoses		Global gene expression data of stage IV CMs (*n* = 57)
Lee et al. 2019 [47]	Multi-variate Cox regression analysis	Pre-operative ctDNA predicts melanoma-specific survival in stage III melanoma		Pre-operative ctDNA from stage III CM patients (*n* = 174)
Mancuso et al. 2021 [48]	Multiple machine learning algorithms (e.g., logistic regression, SVM, decision tree, Gaussian naïve Bayes classifier)	Machine learning algorithm classified early-stage melanoma patients with high and low risk of metastasis; select serum cytokines (e.g., IL-4, GM-CSG, DCD) and Breslow thickness were variables that best predicted metastasis	Accuracy: 80% (Breslow thickness and serum markers model)	Stage I and II melanoma patients (*n* = 323)
Segura et al. 2010 [38]	SAM, KEGG enrichment analysis	18 overexpressed miRNAs were significantly correlated with longer post-recurrence survival	Accuracy: 80.2%	Total RNA of metastatic CMs (*n* = 59)
Sheng et al. 2020 [35]	GO and KEGG enrichment analysis, PPI network analysis	Identified 258 DEGs as potential biomarkers of metastasis		Gene expression data of primary (*n* = 109) and metastatic (*n* = 136) CMs from GEO
Shepelin et al. 2018 [49]	Multiple machine learning algorithms (e.g., SVM, random forest)	Identified 44 characteristic signaling pathways associated with melanoma metastasis	Accuracy: 94% (SVM classifier)	Transcriptomic data of primary and metastatic CMs (*n* = 478) from GEO
Wang et al. 2020 [37]	GO enrichment analysis, PPI network analysis	*CD38* level was a diagnostic factor for CM; high *CD38* expression correlated with higher OS		Gene expression data of CD38 positive CMs from TCGA
Wei et al. 2018 [50]	KEGG and GO enrichment analysis, PPI network analysis, SVM classifier	An SVM predictor for melanoma metastasis had greater than 94% prediction accuracy; 798 DEGs ^1^ were identified	Accuracy: 94.4 to 100%	Gene expression data of primary (*n* = 116) and metastatic (*n* = 296) CMs from GEO and TCGA
Wong et al. 2005 [51]	Nomogram	A nomogram using clinicopathologic information accurately predicted the probability of a positive SLN in melanoma	Accuracy: 69.4%	SLN biopsies (*n* = 979)
Yang et al. 2018 [52]	Two-way hierarchical clustering analysis, SVM classifier, random forest classifier	SVM classifier of a 6 lncRNA signature risk-stratified patients with 85% accuracy	Accuracy: 84.84% (two-way hierarchical clustering), 85.9% (SVM classifier)	lncRNA data of primary CMs (*n* = 376) from TCGA
Zormpas-Petridis et al. 2019 [53]	Spatially constrained-convolution neural network (SC-CNN)	A novel multi-resolution hierarchical framework (SuperCRF) predicted survival based on histology features; SuperCRF had an 12% improvement in accuracy compared to state-of-art SC-CNN cell classifiers	Accuracy: 84.63%	Melanoma H&E slides (*n* = 151)

^1^ AJCC: American Joint Committee on Cancer; AUC: area under the curve; AUROC: area under the receiver operating characteristic; DEG: differentially expressed genes; GO: gene ontology; KEGG: Kyoto Encyclopedia of Genes and Genomes; OS: overall survival; PFS: progression-free survival; RFS: recurrence -free survival; SLN: sentinel lymph node; SVM: support vector machine; TCGA: The Cancer Genome Atlas.

## References

[1] (2021). Cancer Facts & Figures. https://www.cancer.org/research/cancer-facts-statistics/all-cancer-facts-figures/cancer-facts-figures-2021.html.

[2] Robert C., Karaszewska B., Schachter J., Rutkowski P., Mackiewicz A., Stroiakovski D., Lichinitser M., Dummer R., Grange F., Mortier L. (2015). Improved Overall Survival in Melanoma with Combined Dabrafenib and Trametinib. N. Engl. J. Med..

[3] Robert C., Ribas A., Schachter J., Arance A., Grob J.-J., Mortier L., Daud A., Carlino M.S., McNeil C.M., Lotem M. (2019). Pembrolizumab versus Ipilimumab in Advanced Melanoma (KEYNOTE-006): Post-Hoc 5-Year Results from an Open-Label, Multicentre, Randomised, Controlled, Phase 3 Study. Lancet Oncol..

[4] de Gruijl F.R. (2002). Photocarcinogenesis: UVA vs. UVB Radiation. Ski. Pharm. Appl Ski. Physiol..

[5] Rastrelli M., Tropea S., Rossi C.R., Alaibac M. (2014). Melanoma: Epidemiology, Risk Factors, Pathogenesis, Diagnosis and Classification. In Vivo.

[6] Albino A.P., Le Strange R., Oliff A.I., Furth M.E., Old L.J. (1984). Transforming Ras Genes from Human Melanoma: A Manifestation of Tumour Heterogeneity?. Nature.

[7] Hussussian C.J., Struewing J.P., Goldstein A.M., Higgins P.A.T., Ally D.S., Sheahan M.D., Clark W.H., Tucker M.A., Dracopoli N.C. (1994). Germline P16 Mutations in Familial Melanoma. Nat. Genet..

[8] Ghosh P., Chin L. (2009). Genetics and Genomics of Melanoma. Expert Rev. Derm..

[9] Davies H., Bignell G.R., Cox C., Stephens P., Edkins S., Clegg S., Teague J., Woffendin H., Garnett M.J., Bottomley W. (2002). Mutations of the BRAF Gene in Human Cancer. Nature.

[10] Chapman P.B., Hauschild A., Robert C., Haanen J.B., Ascierto P., Larkin J., Dummer R., Garbe C., Testori A., Maio M. (2011). Improved Survival with Vemurafenib in Melanoma with BRAF V600E Mutation. N. Engl. J. Med..

[11] Hauschild A., Grob J.-J., Demidov L.V., Jouary T., Gutzmer R., Millward M., Rutkowski P., Blank C.U., Miller W.H., Kaempgen E. (2012). Dabrafenib in BRAF-Mutated Metastatic Melanoma: A Multicentre, Open-Label, Phase 3 Randomised Controlled Trial. Lancet.

[12] Barba M., Czosnek H., Hadidi A. (2014). Historical Perspective, Development and Applications of Next-Generation Sequencing in Plant Virology. Viruses.

[13] Hodis E., Watson I.R., Kryukov G.V., Arold S.T., Imielinski M., Theurillat J.-P., Nickerson E., Auclair D., Li L., Place C. (2012). A Landscape of Driver Mutations in Melanoma. Cell.

[14] Krauthammer M., Kong Y., Ha B.H., Evans P., Bacchiocchi A., McCusker J.P., Cheng E., Davis M.J., Goh G., Choi M. (2012). Exome Sequencing Identifies Recurrent Somatic RAC1 Mutations in Melanoma. Nat. Genet..

[15] The Cancer Genome Atlas Program—National Cancer Institute. https://www.cancer.gov/about-nci/organization/ccg/research/structural-genomics/tcga.

[16] Hayward N.K., Wilmott J.S., Waddell N., Johansson P.A., Field M.A., Nones K., Patch A.-M., Kakavand H., Alexandrov L.B., Burke H. (2017). Whole-Genome Landscapes of Major Melanoma Subtypes. Nature.

[17] Trevarton A., Mann M., Knapp C., Araki H., Wren J., Stones-Havas S., Black M., Print C. (2013). MelanomaDB: A Web Tool for Integrative Analysis of Melanoma Genomic Information to Identify Disease-Associated Molecular Pathways. Front. Oncol..

[18] Papadodima O., Kontogianni G., Piroti G., Maglogiannis I., Chatziioannou A. (2019). Genomics of Cutaneous Melanoma: Focus on next-Generation Sequencing Approaches and Bioinformatics. J. Transl. Genet. Genom..

[19] Dummer R., Hauschild A., Santinami M., Atkinson V., Mandalà M., Kirkwood J.M., Chiarion Sileni V., Larkin J., Nyakas M., Dutriaux C. (2020). Five-Year Analysis of Adjuvant Dabrafenib plus Trametinib in Stage III Melanoma. N. Engl. J. Med..

[20] Eggermont A.M.M., Blank C.U., Mandala M., Long G.V., Atkinson V., Dalle S., Haydon A., Lichinitser M., Khattak A., Carlino M.S. (2018). Adjuvant Pembrolizumab versus Placebo in Resected Stage III Melanoma. N. Engl. J. Med..

[21] Weber J., Mandala M., Del Vecchio M., Gogas H.J., Arance A.M., Cowey C.L., Dalle S., Schenker M., Chiarion-Sileni V., Marquez-Rodas I. (2017). Adjuvant Nivolumab versus Ipilimumab in Resected Stage III or IV Melanoma. N. Engl. J. Med..

[22] Long G.V., Hauschild A., Santinami M., Atkinson V., Mandalà M., Chiarion-Sileni V., Larkin J., Nyakas M., Dutriaux C., Haydon A. (2017). Adjuvant Dabrafenib plus Trametinib in Stage III BRAF-Mutated Melanoma. N. Engl. J. Med..

[23] Bensimon A.G., Zhou Z.-Y., Jenkins M., Song Y., Gao W., Signorovitch J., Krepler C., Liu F.X., Wang J., Aguiar-Ibáñez R. (2019). Cost-Effectiveness of Pembrolizumab for the Adjuvant Treatment of Resected High-Risk Stage III Melanoma in the United States. J. Med. Econ..

[24] Coit D.G., Thompson J.A., Algazi A., Andtbacka R., Bichakjian C.K., Carson W.E., Daniels G.A., DiMaio D., Ernstoff M., Fields R.C. (2016). Melanoma, Version 2.2016, NCCN Clinical Practice Guidelines in Oncology. J. Natl. Compr. Cancer Netw..

[25] Gershenwald J.E., Scolyer R.A., Hess K.R., Sondak V.K., Long G.V., Ross M.I., Lazar A.J., Faries M.B., Kirkwood J.M., McArthur G.A. (2017). Melanoma Staging: Evidence-Based Changes in the American Joint Committee on Cancer Eighth Edition Cancer Staging Manual. CA Cancer J. Clin..

[26] Poklepovic A.S., Luke J.J. (2020). Considering Adjuvant Therapy for Stage II Melanoma. Cancer.

[27] Jönsson G., Busch C., Knappskog S., Geisler J., Miletic H., Ringnér M., Lillehaug J.R., Borg A., Lønning P.E. (2010). Gene Expression Profiling-Based Identification of Molecular Subtypes in Stage IV Melanomas with Different Clinical Outcome. Clin. Cancer Res..

[28] Gerami P., Cook R.W., Wilkinson J., Russell M.C., Dhillon N., Amaria R.N., Gonzalez R., Lyle S., Johnson C.E., Oelschlager K.M. (2015). Development of a Prognostic Genetic Signature to Predict the Metastatic Risk Associated with Cutaneous Melanoma. Clin. Cancer Res..

[29] Gerami P., Cook R.W., Russell M.C., Wilkinson J., Amaria R.N., Gonzalez R., Lyle S., Jackson G.L., Greisinger A.J., Johnson C.E. (2015). Gene Expression Profiling for Molecular Staging of Cutaneous Melanoma in Patients Undergoing Sentinel Lymph Node Biopsy. J. Am. Acad. Dermatol..

[30] Kovarik C.L., Chu E.Y., Adamson A.S. (2020). Gene Expression Profile Testing for Thin Melanoma: Evidence to Support Clinical Use Remains Thin. JAMA Dermatol..

[31] Zager J.S., Gastman B.R., Leachman S., Gonzalez R.C., Fleming M.D., Ferris L.K., Ho J., Miller A.R., Cook R.W., Covington K.R. (2018). Performance of a Prognostic 31-Gene Expression Profile in an Independent Cohort of 523 Cutaneous Melanoma Patients. BMC Cancer.

[32] Gastman B.R., Gerami P., Kurley S.J., Cook R.W., Leachman S., Vetto J.T. (2019). Identification of Patients at Risk of Metastasis Using a Prognostic 31-Gene Expression Profile in Subpopulations of Melanoma Patients with Favorable Outcomes by Standard Criteria. J. Am. Acad. Dermatol..

[33] Marchetti M.A., Coit D.G., Dusza S.W., Yu A., McLean L., Hu Y., Nanda J.K., Matsoukas K., Mancebo S.E., Bartlett E.K. (2020). Performance of Gene Expression Profile Tests for Prognosis in Patients With Localized Cutaneous Melanoma: A Systematic Review and Meta-Analysis. JAMA Derm..

[34] Hu Y., Briggs A., Marchetti M.A., Ariyan C.E., Coit D.G., Bartlett E.K. (2020). Cost-Benefit Implication of Gene Expression Profiling and Adjuvant Therapy in Stage IIIA Melanoma. J. Am. Coll. Surg..

[35] Sheng Z., Han W., Huang B., Shen G. (2020). Screening and Identification of Potential Prognostic Biomarkers in Metastatic Skin Cutaneous Melanoma by Bioinformatics Analysis. J. Cell. Mol. Med..

[36] Jiang J., Liu C., Xu G., Liang T., Yu C., Liao S., Zhang Z., Lu Z., Wang Z., Chen J. (2021). Identification of Hub Genes Associated With Melanoma Development by Comprehensive Bioinformatics Analysis. Front. Oncol..

[37] Wang R., Shao X., Zheng J., Saci A., Qian X., Pak I., Roy A., Bello A., Rizzo J.I., Hosein F. (2020). A Machine-Learning Approach to Identify a Prognostic Cytokine Signature That Is Associated with Nivolumab Clearance in Patients with Advanced Melanoma. Clin. Pharmacol. Ther..

[38] Segura M.F., Belitskaya-Lévy I., Rose A.E., Zakrzewski J., Gaziel A., Hanniford D., Darvishian F., Berman R.S., Shapiro R.L., Pavlick A.C. (2010). Melanoma MicroRNA Signature Predicts Post-Recurrence Survival. Clin. Cancer Res..

[39] Arora C., Kaur D., Lathwal A., Raghava G.P.S. (2020). Risk Prediction in Cutaneous Melanoma Patients from Their Clinico-Pathological Features: Superiority of Clinical Data over Gene Expression Data. Heliyon.

[40] Bellomo D., Arias-Mejias S.M., Ramana C., Heim J.B., Quattrocchi E., Sominidi-Damodaran S., Bridges A.G., Lehman J.S., Hieken T.J., Jakub J.W. (2020). Model Combining Tumor Molecular and Clinicopathologic Risk Factors Predicts Sentinel Lymph Node Metastasis in Primary Cutaneous Melanoma. JCO Precis. Oncol..

[41] Brinker T.J., Kiehl L., Schmitt M., Jutzi T.B., Krieghoff-Henning E.I., Krahl D., Kutzner H., Gholam P., Haferkamp S., Klode J. (2021). Deep Learning Approach to Predict Sentinel Lymph Node Status Directly from Routine Histology of Primary Melanoma Tumours. Eur. J. Cancer.

[42] Cheng Y., Lu J., Chen G., Ardekani G.S., Rotte A., Martinka M., Xu X., McElwee K.J., Zhang G., Zhou Y. (2015). Stage-Specific Prognostic Biomarkers in Melanoma. Oncotarget.

[43] Farrow N.E., Holl E.K., Jung J., Gao J., Jung S.-H., Al-Rohil R.N., Selim M.A., Mosca P.J., Ollila D.W., Antonia S.J. (2021). Characterization of Sentinel Lymph Node Immune Signatures and Implications for Risk Stratification for Adjuvant Therapy in Melanoma. Ann. Surg. Oncol..

[44] Garg M., Couturier D.-L., Nsengimana J., Fonseca N.A., Wongchenko M., Yan Y., Lauss M., Jönsson G.B., Newton-Bishop J., Parkinson C. (2021). Tumour Gene Expression Signature in Primary Melanoma Predicts Long-Term Outcomes. Nat. Commun..

[45] Huang M., Zhang Y., Ou X., Wang C., Wang X., Qin B., Zhang Q., Yu J., Zhang J., Yu J. (2021). M5C-Related Signatures for Predicting Prognosis in Cutaneous Melanoma with Machine Learning. J. Oncol..

[46] Johannet P., Coudray N., Donnelly D.M., Jour G., Illa-Bochaca I., Xia Y., Johnson D.B., Wheless L., Patrinely J.R., Nomikou S. (2021). Using Machine Learning Algorithms to Predict Immunotherapy Response in Patients with Advanced Melanoma. Clin. Cancer Res..

[47] Lee J.H., Saw R.P., Thompson J.F., Lo S., Spillane A.J., Shannon K.F., Stretch J.R., Howle J., Menzies A.M., Carlino M.S. (2019). Pre-Operative CtDNA Predicts Survival in High-Risk Stage III Cutaneous Melanoma Patients. Ann. Oncol..

[48] Mancuso F., Lage S., Rasero J., Díaz-Ramón J.L., Apraiz A., Pérez-Yarza G., Ezkurra P.A., Penas C., Sánchez-Diez A., García-Vazquez M.D. (2020). Serum Markers Improve Current Prediction of Metastasis Development in Early-Stage Melanoma Patients: A Machine Learning-Based Study. Mol. Oncol..

[49] Shepelin D., Korzinkin M., Vanyushina A., Aliper A., Borisov N., Vasilov R., Zhukov N., Sokov D., Prassolov V., Gaifullin N. (2015). Molecular Pathway Activation Features Linked with Transition from Normal Skin to Primary and Metastatic Melanomas in Human. Oncotarget.

[50] Wei D. (2018). A Multigene Support Vector Machine Predictor for Metastasis of Cutaneous Melanoma. Mol. Med. Rep..

[51] Wong S.L., Kattan M.W., McMasters K.M., Coit D.G. (2005). A Nomogram That Predicts the Presence of Sentinel Node Metastasis in Melanoma With Better Discrimination Than the American Joint Committee on CancerStaging System. Ann. Surg. Oncol..

[52] Yang S., Xu J., Zeng X. (2018). A Six-Long Non-Coding RNA Signature Predicts Prognosis in Melanoma Patients. Int. J. Oncol..

[53] Zormpas-Petridis K., Failmezger H., Raza S.E.A., Roxanis I., Jamin Y., Yuan Y. (2019). Superpixel-Based Conditional Random Fields (SuperCRF): Incorporating Global and Local Context for Enhanced Deep Learning in Melanoma Histopathology. Front. Oncol..

[54] Piñero A., Canteras M., Ortiz E., Martínez-Barba E., Parrilla P. (2008). Validation of a Nomogram to Predict the Presence of Sentinel Lymph Node Metastases in Melanoma. Ann. Surg. Oncol..

[55] Pasquali S., Mocellin S., Campana L.G., Vecchiato A., Bonandini E., Montesco M.C., Santarcangelo S., Zavagno G., Nitti D., Rossi C.R. (2011). Maximizing the Clinical Usefulness of a Nomogram to Select Patients Candidate to Sentinel Node Biopsy for Cutaneous Melanoma. Eur. J. Surg. Oncol..

[56] Woods J.F.C., De Marchi J.A., Lowery A.J., Hill A.D.K. (2015). Validation of a Nomogram Predicting Sentinel Lymph Node Status in Melanoma in an Irish Population. Ir. J. Med. Sci..

[57] Schrider D.R., Kern A.D. (2018). Supervised Machine Learning for Population Genetics: A New Paradigm. Trends Genet..

[58] Libbrecht M.W., Noble W.S. (2015). Machine Learning Applications in Genetics and Genomics. Nat. Rev. Genet..

[59] Thomsen K., Iversen L., Titlestad T.L., Winther O. (2020). Systematic Review of Machine Learning for Diagnosis and Prognosis in Dermatology. J. Dermatol. Treat..

